# Triage for Malnutrition Risk among Pediatric and Adolescent Outpatients with Cystic Fibrosis, Using a Disease-Specific Tool

**DOI:** 10.3390/children7120269

**Published:** 2020-12-04

**Authors:** Dimitrios Poulimeneas, Maria G. Grammatikopoulou, Argyri Petrocheilou, Athanasios G. Kaditis, Tonia Vassilakou

**Affiliations:** 1 Department of Public Health Policy, School of Public Health, University of West Attica, 196, Alexandras Avenue, GR-11521 Athens, Greece; dpoul@hua.gr; 2 Department of Nutrition and Dietetics, Harokopio University, E. Venizelou 70, GR-17671 Athens, Greece; 3 Department of Nutritional Sciences & Dietetics, Alexander Campus, International Hellenic University, GR-57001 Thessaloniki, Greece; maria@ihu.gr; 4 Cystic Fibrosis Department, Agia Sophia Children’s Hospital, Thivon 1, GR-11527 Athens, Greece; apetroch@gmail.com (A.P.); kaditia@hotmail.com (A.G.K.); 5 Division of Pediatric Pulmonology and Sleep Disorders Laboratory, First Department of Pediatrics, National and Kapodistrian University of Athens School of Medicine and Aghia Sophia Children’s Hospital, Thivon 1, GR-11527 Athens, Greece

**Keywords:** children, pulmonary disease, forced expiratory volume, pulmonary infection, nutritional assessment, screening, underweight, pulmonary function, pancreatic insufficiency, PERT

## Abstract

Malnutrition prevails in considerable proportions of children with Cystic Fibrosis (CF), and is often associated with adverse outcomes. For this, routine screening for malnutrition is pivotal. In the present cross-sectional study, we aimed to assess the risk for malnutrition in pediatric outpatients with CF. A total of 76 outpatients (44 girls, 11.9 ± 3.9 years old, 39.5% adolescents) were recruited and anthropometric, clinical, dietary and respiratory measures were collected. All outpatients were screened for malnutrition risk with a validated disease-specific instrument. Most children exhibited a low risk for malnutrition (78.9%), whereas none of the participants were characterized as having a high malnutrition risk. In the total sample, malnutrition risk was positively associated with age (*r* = 0.369, *p* = 0.001), and inversely related to the body mass index (*r* = −0.684, *p* < 0.001), height z-score (*r* = −0.264, *p* = 0.021), and forced expiratory volume (FEV_1_%, *r* = −0.616, *p* < 0.001). Those classified as having a low malnutrition risk were younger (*p* = 0.004), heavier (*p* < 0.001) and taller (*p* = 0.009) than their counterparts with a moderate risk. On the other hand, patients in the moderate risk group were more likely pubertal (*p* = 0.034), with a reduced mid-upper arm fat area (*p* = 0.011), and worse pulmonary function (*p* < 0.001). Interestingly, none of the children attaining ideal body weight were classified as having a moderate malnutrition. risk, whereas 37.5% of the patients allocated at the moderate risk group exhibited physiological lung function. In this cohort of outpatients with CF that were predominantly well-nourished and attained physiological lung function, malnutrition risk was identified only in small proportions of the sample. Our data support that patients that are older, pubertal, or have diminished fat mass are at greater risk for malnutrition.

## 1. Introduction

Cystic fibrosis (CF) is a chronic condition characterized by an increased risk of malnutrition. Poor nutritional intake due to increased energy loss and demands, inadequate caloric intake, malabsorption and maldigestion, exacerbations of the disease, genetic susceptibility and the often underlying infections, increase the risk of malnutrition among patients with CF [1,2,3,4,5,6]. Between adults and children, the latter appear to be more vulnerable to the development of malnutrition, due to their yet underdeveloped system, further increasing their nutrient demands and susceptibility to infection [5]. Nevertheless, malnutrition during childhood has also been associated with an increased malnutrition risk during adult years and a greater probability of requiring lung transplantation [7].

Although several research projects have revealed a high degree of malnourishment among children with CF [8,9,10], early identification of such cases, through screening, is pivotal for early intervention and improved outcomes. In parallel, today, malnutrition identification practice is mainly reliant on anthropometric data, the subjective assessment and clinical judgement of each pediatrician, with the mainstay of this approach being biased by the often-inadequate nutritional expertise of pediatricians [11]. Severe cases of malnutrition might be more evident to the untrained eye, however, children at risk, or milder forms of malnutrition, often remain undiagnosed [12]. With this in mind, a variety of screening tools have been developed for pediatric patients, most of which can be used among hospitalized children, with the majority exhibiting good sensitivity and specificity [13].

With regards to CF however, disease-specific screening tools for malnutrition are limited, especially for pediatric patients, with only two existing in the literature to date, the first being published during 2008 [14] and the more recent one presented in the year 2016 [15]. The use of malnutrition screening tools specifically for each underlying disease, in this case CF, is important, as the pathophysiology of the disease contributing to the development of malnutrition is considered, all factors contributing to the development of malnutrition in the condition are accounted for and a more accurate triage of patients is enabled.

With this in mind, the aim of the present study was to assess the risk for malnutrition among pediatric patients with CF, using a disease-specific tool.

## 2. Materials and Methods

### 2.1. Sample Recruitment

The GreeCF study is an observational study, aiming to assess the relationship between growth indices, clinical and dietary parameters among school-aged children with CF. The study was conducted during the last quarter of 2015, in the outpatient Clinic for CF, situated in Aghia Sophia Children’s Hospital, in metropolitan Athens, Greece. Details concerning the recruitment have been previously reported [10,16]. All children were screened for eligibility, during their routine appointment at the clinic. Inclusion criteria involved (1) having an age between 6–18 years, (2) having a confirmed CF diagnosis and (3) willingness to participate. Exclusion criteria included (1) being < 6 years old, or being an adult, (2) having a concurrent disease affecting growth, and (3) refusal of the parents/guardians to provide informed consent for participation. A total of 114 children/adolescents with CF met the inclusion criteria. For the purposes of the present paper, we analyzed the data of 76 patients (response rate 66.7%, mean age 11.9 ± 3.9 years old, 44 girls, 39.5% adolescents) with complete answers on all measures required for the analyses.

The parents/guardians of all patients provided informed written consent prior to participation. The study was conducted in accordance with the Declaration of Helsinki, and the protocol was approved by the Ethics Committees of both Aghia Sophia Children’s Hospital, and University of West Attica (reference number 16084/14-07-15).

### 2.2. Anthropometric Measures

Anthropometry was conducted during morning hours, with patients wearing light clothing and having bare feet, by the same experienced dietician (DP). Body weight was measured at the nearest 0.1 kg (SECA 874 portable digital scale, Hamburg, Germany) and height to the nearest 0.5 cm (SECA 214 portable stadiometer, Hamburg, Germany), and then body mass index (BMI) was computed. Height-for-age (HAZ) and BMI-for age (BAZ) z-scores were calculated for each patient, based on the Centers for Disease Control (CDC) growth charts [17]. According to the BAZ, patients were classified as underweight (BAZ < −2.0), or normal body weight (−2.0 ≤ BAZ < 1.0), overweight or obese (BAZ ≥ 1.0, and BAZ ≥ 2.0, respectively). CF-specific classification was used to assess ideal body weight (IBW) (BAZ ≥ 0.0) and possible nutritional failure (BAZ < −1.04). Children with an HAZ < −2.0 were considered chronically malnourished (stunting).

On the left side of the body, mid-upper arm circumference (MUAC) was measured at the mid-point between the olecranon process and the acromium, while triceps’ skinfold thickness (TSF) values were taken three times (Harpenden Skinfold Calipers, Batty International, Burgess Hill, England) in order to attain a median value. MUAC, TSF, mid upper arm muscle (MUAMA) and fat area (MUAFA) z-scores were calculated according to the NHANES III survey [18].

Dietary intake was assessed at the day of the visit, through a multiple-pass, 24-h dietary recall, by the same experienced dietician (D.P.). For children aged below 9 years old, parental consensus during the interview was sought. Recall data were then analyzed for total energy intake (TEI) using the Food Processor software (ESHA, Portland, Oregon). TEI was then converted in % of the recommended daily intake.

### 2.3. Patients’ Medical History, Physical and Clinical Parameters and CF-Related Comorbidities

Individual medical records were searched for age at diagnosis and diagnostic criteria, presence of pancreatic insufficiency (PI), CF-related diabetes (CFRD), intermittent and/or chronic infection by pathogens (*Pseudomonas Aeruginosa*, methicillin resistant *Staphylococcus Aureus*, *Burkholderia cepacia*), enteral feeding, and serum albumin levels. Pulmonary function was assessed during the day of each patient’s visit to the clinic, through the Forced Expiratory Volume at 1 s (FEV_1_). Pulmonary function was compared against predicted values (FEV_1_%) using the equations suggested by Wang et al. [19] (for boys aged 6–17 years old and girls 6–15 years old) and Hankinson et al. (for boys aged 18 years old and girls exceeding the 16 years of age) [20].

Measurements of weight and height collected during previous visits were also recorded to evaluate impaired weight gain and/or weight loss, and failure to thrive. Puberty was defined after physical examination (and/or menarche in girls) by the team of medical doctors; no specific data regarding the pubertal stages of the patients were recorded.

### 2.4. Malnutrition Risk

Malnutrition Risk was assessed by the validated CF-specific tool, developed by dos Santos Simon and associates [15]. In more detail, this tool assigns ratings based on 10 risk factors including: (i) BMI status, (ii) presence of PI and (iii) CFRD, (iv) colonization by specific pathogens, (v) reduced dietary intake, (vi) impaired weight gain or involuntary weight loss, (vii) impaired height gain, (viii) enteral feeding, (ix) impaired lung function as evidenced by FEV_1_%, and (x) low serum albumin levels. A composite score is then computed by summing up the ratings of each risk factor. The score ranges from 0 to 14, with higher values indicative of higher nutritional risk. Furthermore, ratings between 0–3 indicate low malnutrition risk, ratings of 4–7 suggest medium risk, whereas a score ≥ 8 is indicative of increased risk for malnutrition [15].

### 2.5. Statistical Analyses

Data distribution was visually explored with Q-Q plots; all continuous variables but age at diagnosis (months) were normally distributed. Therefore, continuous variables are presented as means ± standard deviation, whereas age at diagnosis is presented as a median with its respective interquartile ranges (1st and 3rd IQR). Categorical variables are presented as relative frequencies and percentages. Differences between continuous variables were assessed with independent *t*-test (or Mann–Whitney test for age at diagnosis). Chi-square or the Fisher’s exact test was employed to explore differences between categorical variables. Association between continuous variables was examined through Spearman’s r correlation co-efficient. The level of significance was set at *α* = 0.05. All analyses were performed with SPSS version 25.0 (IBM, SPSS Inc., Chicago, IL, USA). 

## 3. Results

### 3.1. Assessment of Malnutrition Risk

A total of 32 boys and 44 girls were recruited for the purposes of this study (aged 11.9 ± 3.9 years old, 39.5% adolescents). Their mean BAZ was 0.05 ± 1.13, whereas their mean HAZ was −0.28 ± 1.09. Referring to their pulmonary function, patients exhibited a mean FEV1% of 98.0 ± 19.8%.

Risk factors for malnutrition, and malnutrition risk are presented in Table 1. None of the patients exhibited suboptimal albumin levels, or received enteral feedings. Only a small minority of the examined children failed to reach linear growth, or had impaired lung function. On the contrary, most children (59.2%) attained IBW for CF, and reached the recommended targets for energy intake (71.1%). A great proportion of participants had pancreatic insufficiency (86.8%), whereas none of the children were on enteral nutrition or exhibited low serum albumin levels. Most patients exhibited a low risk for malnutrition (78.9%), whereas none of the participants were characterized as having high malnutrition risk. No significant differences between the examined risk factors, or total malnutrition risk and malnutrition risk categories was observed according to the sex of the participants (*p* > 0.05 for all comparisons, data not shown).

### 3.2. Factors Associated with Malnutrition Risk

In the total sample, a moderate positive association was noted between malnutrition risk and age (*r* = 0.369, *p* = 0.001). Malnutrition risk was inversely associated with BAZ (*r* = −0.684, *p* < 0.001), HAZ (*r* = −0.264, *p* = 0.021), and FEV1% (*r* = −0.616, *p* < 0.001). Referring to arm anthropometrics, malnutrition risk was not associated with TSF, MUAC, MUAMA or MUAFA z-scores (*p* > 0.05 for all comparisons). When the aforementioned indices were examined according to sex, similar results were revealed, with the exception of age. In further detail, malnutrition risk did not significantly associate with the age of boys (data not shown).

When patients were stratified according to malnutrition risk, several differences between the groups were identified (Table 2). Patients classified as having a low malnutrition risk were younger (*p* = 0.004), heavier (*p* < 0.001) and taller (*p* = 0.009) than their moderate-risk counterparts. Significantly more patients in the medium-risk group were in puberty (*p* = 0.034), whereas patients in the moderate-risk group exhibited smaller mean mid upper arm fat area (*p* = 0.011), and overall worse pulmonary function (*p* < 0.001).

None of the other clinical parameters were associated with any of the nutritional risk strata. Interestingly, among the children attaining ideal body weight, none were categorized as being of moderate risk for malnutrition. On the other hand, 37.5% of the participants stratified as being at moderate risk for malnutrition exhibited physiological lung function.

## 4. Discussion

In the present study, participants with CF were predominantly well-nourished and exhibited adequate physiological lung function, with a moderate risk for malnutrition prevailing only in small proportions (1/5) of the sample, and none of the patients were classified as being of high malnutrition risk. Malnutrition risk was inversely associated with patients’ anthropometry and lung function. Although none of the patients attaining ideal body weight for CF were at nutritional risk, this was not the case for physiological lung function, with 38% of the children with FEV_1_ > 90% being allocated in the moderate risk for malnutrition group.

Throughout the literature, BMI z-scores have been extensively employed to identify malnutrition in pediatric CF patients, using a variety of cut-offs (Table 3). Most of the studies suggest that attaining a BMI below the 10th percentile (roughly corresponding to a z-score < −1.0) is indicative of malnutrition. With this in mind, published data suggest that malnutrition prevails in 12–74% of pediatric patients with CF [21,22,23,24,25,26]. Some earlier studies using the stricter cut-off of the 15th BMI percentile to identify malnutrition support similar findings, with malnutrition being diagnosed in 20–30% of pediatric patients [27,28,29,30]. Nevertheless, very few studies have examined the risk for malnutrition among pediatric patients with CF. In the validation study of the screening tool employed in the present study, dos Santos Simon and colleagues [15] reported a moderate malnutrition risk in 36.6% of their sample and a high malnutrition risk at 7.3% of their pediatric patients.

In the present analysis, patients at risk for malnutrition were shorter and exhibited a lower BMI than their counterparts with low-malnutrition risk. While malnutrition is a known effector impeding linear growth, weight and height measures have also been critiqued for not providing early signs of malnutrition in children with CF [31].

A similar case might also apply for FEV_1_. Consistent with the existing literature [32], patients at malnutrition risk exhibited worse pulmonary function. Notably, even though the moderate malnutrition risk group demonstrated an impaired mean pulmonary function than the low-risk group, 38% of the patients with moderate malnutrition risk exhibited an adequate lung function (predicted FEV_1_ ≥ 90%). At the same time, 1/5 of the children with low malnutrition risk demonstrated impaired lung function (predicted FEV_1_ < 90%). These observations may indicate that in response to malnutrition, lung function is delayed, or that FEV_1_ measures may not be sensitive enough during the early phase and signs of malnutrition (given that in a large proportion of the patients, malnutrition risk was “masked” under adequate lung function). For this reason, triage for malnutrition using CF-specific tools is required in all cases and one should not rely on anthropometric indices or pulmonary function alone. It may also be that these measures are more sensitive in identifying malnutrition risk when their secular trends are taken into account, such as when using CF-specific screening tools for malnutrition, like in the present study.

Other risk factors for malnutrition identified in the present study include ascending age, puberty, and diminished fat mass. In our sample, age was positively correlated with malnutrition risk, a finding more profound among girl participants. Regardless of sex, BMI has been previously shown to decline with age [27], while, on the other hand, age has been associated with malnutrition [24]. The fact that this association was more apparent among girls may be attributed to the worse influence of the disease in the girls as compared to the boys, as previously documented in the literature [33,34].

With regards to puberty, it remains a grey area in CF-research. Girls with CF have been reported to enter puberty with a two year delay as compared to their healthy counterparts [35,36]. In parallel, a delay in reaching peak height velocity in adolescence has been reported to take place [36], and for this, a greater proportion of adolescents with CF are underweight as compared to the children [16]. This delay in puberty has also been reported to influence bone health and mineralization [37], making patients with CF more vulnerable to fractures and osteoporosis [38].

With regards to the relationship between low fat mass and malnutrition risk, very few studies have assessed body composition among children with CF. Although a variety of tools have been proposed for the assessment of body composition in CF [39], the majority of studies have used adult samples. In parallel, a consensus on body composition measurements, diagnoses and thresholds is required to attain a uniform core set of outcomes [40]. Case-control studies have revealed a greater visceral adipose tissue mass among patients with CF as compared to healthy controls, and this was associated with poorer diet quality [41]. In parallel, according to many cross-sectional studies, reduced fat-free mass has been associated with reduced pulmonary function assessed by FEV_1_ and frequency of exacerbations in both children and adults with CF [42,43,44]. All studies agree that fat-free mass is more sensitive than BMI for assessing malnutrition and compromised pulmonary function. Overall, our findings, combined with the current literature, call for the need for routine measurements of multiple auxological indices (body height and weight, along with body composition measures) in children and adolescents with CF, in order to attain timely assessments of malnutrition risk.

The limitations of the present study include its cross-sectional design, not allowing for the establishment of causality in the observed relationships. The sample might appear as relatively small, however, sampling was performed in the largest CF center in Greece, located in the capital of Greece providing CF-care to a large population with CF. The inclusion of younger children (aged < 6 years old) in our sample may have provided more robust results, given that early-life malnutrition impairs lung function, and this impairment is persistent for over a decade [45]. Although a greater malnutrition risk was identified during adolescence, no data were recorded with regards to the pubertal stage. Stratification by pubertal stage might have provided further insight into the observed relationships, an analysis we were unable to perform and that future studies should elaborate on. Cystic Fibrosis Transmembrane Regulator (CFTR) modulator therapy commenced shortly after the present study was conducted [46], hence no data were reported on the association of malnutrition risk and the specific therapy. This evidence gap should be further examined by future research, given the positive effects of CFTR modulators in anthropometric indices of individuals with CF [47]. Furthermore, no data concerning biochemical parameters and malnutrition risk were recorded. It is highly possible that serum lipid levels or circulating levels of other nutrients might have produced significant associations with malnutrition risk, however, we did not want to cherry-pick associations that were not predefined in advance. On the other hand, the present study is one of the few that have assessed malnutrition risk in the pediatric CF population, further highlighting risk factors for malnutrition that may be masked under optimal clinical outcomes (such as physiological lung function).

## 5. Conclusions

In this cohort of predominantly well-nourished patients with CF, 1/5 of the patients were at risk for malnutrition, with those that were older, pubertal or with diminished fat-mass being at higher risk. Screening for malnutrition risk in pediatric patients with CF is important, although often neglected. Even in populations with a low prevalence of malnutrition, the risk for malnutrition might be elevated, highlighting the need for more frequent assessments and nutrition interventions.

## Figures and Tables

**Table 1:** Prevalence of malnutrition risk factors, and total malnutrition risk score among participating children, according to the Nutrition Screening Tool for Pediatric Patients with Cystic Fibrosis [15] (*N* = 76).

Risk Factor		*n* (%) *
1	BMI < 50th percentile	31 (40.8)
	BMI < 10th percentile	10 (13.2)
2	Pancreatic Insufficiency	66 (86.8)
3	*Pseudomonas, Burkholderia cepacia* or MRSA colonization	29 (38.2)
4	Dietary Intake < 100% RDA	22 (28.9)
5	Weight gain less than minimum, zero weight gain or weight loss	6 (7.9)
6	Height gain less than minimum, or zero height gain	4 (5.3)
7	Enteral feeding	0 (0.0)
8	CFRD	4 (5.3)
9	FEV_1_% (< 80% predicted)	13 (17.1)
10	Serum albumin < 3.5 mg/dL	0 (0.0)
Mean total Malnutrition Score		2.43 ± 1.52
	Patients at low risk for malnutrition (*n*, %)	60 (78.9)
	Patients at average risk for malnutrition (*n*, %)	16 (21.1)
	Patients at high risk for malnutrition (*n*, %)	0 (0.0)

BMI, body mass index; CFRD, Cystic-fibrosis-related diabetes; FEV_1_, forced expiratory volume at 1 s; MRSA, methicillin-resistant *Staphylococcus aureus*; PI, pancreatic insufficiency; RDA, recommended dietary allowance; * Values presented as *n* (%), or mean ± standard deviation.

**Table 2:** Associations between anthropometry and clinical parameters, by malnutrition risk strata (*N* = 76).

Variable	Malnutrition Risk		*p*-Value
	Low (*n* = 60)	Medium (*n* = 16)	
Sex (% girls)	58.3	56.3	0.881
Age (years)	11.2 ± 3.8	14.4 ± 3.4	0.004
Adolescence (%)	33.3	62.5	0.034
BAZ	0.41 ± 0.92	−1.28 ± 0.78	<0.001
Underweight (%)	3.3	18.8	0.027
Nutritional Failure (%)	8.3	56.3	<0.001
Ideal Body Weight (%)	75.0	0.0	<0.001
HAZ	−0.10 ± 1.04	−0.93 ± 1.03	0.009
Stunting (%)	3.3	12.5	0.145
MUAC*z*	−0.41 ± 0.96	−0.46 ± 1.05	0.885
TSF*z*	1.56 ± 0.80	1.82 ± 1.07	0.371
MUAMA*z*	0.16 ± 2.77	−0.29 ± 2.54	0.562
MUAFA*z*	1.24 ± 1.45	0.23 ± 0.90	0.011
FEV_1_%	103.4 ± 15.4	77.7 ± 21.8	<0.001
Dietary Intake (% RDA)	137 ± 49	125 ± 42	0.384
FEV_1_ > 90% (%)	80.4	37.5	0.001
Homozygote *F508*del (%)	43.3	31.3	0.382
Age at diagnosis (months)	3.5 (1.0, 8.8)	5.0 (1.0, 9.5)	0.627
Meconium Ileus (%)	16.7	25.0	0.445
Diagnosis by NBS (%)	13.3	6.3	0.436

BAZ, body mass index for age; FEV_1_, forced expiratory volume at 1 s; HAZ, height-for age; MUAC, mid upper arm circumference; MUAFA, mid upper arm fat area; MUAMA, mid upper arm muscle area; NBS, new-born screening; RDA, recommended dietary allowance; TSF, triceps skinfold.

**Table 3:** The existing literature on the prevalence of malnutrition among children and adolescents with CF.

First Author	Country	Population		Cut-off Used for Malnutrition Diagnosis	Prevalence of Malnutrition (%)
		*N* (% Girls)	Age (Years)		
Barni [24]	Brasil	73 (55%)	25.6 ± 7.3	BMI < 10th percentile	24.7
Chaves [26]	Brasil	48 (NR)	10.8 ± 3.3	BMI < 10th percentile	29
Isa [23]	Bahrain	47 (43%)	<18	BMI < 10th percentile	22.1
Kilinc [22]	Turkey	143 (47%)	0–18	BMI < 10th percentile	74
Lucidi [28]	Italy	82 (49%)	13 (5–30)	BMI < 15th percentile	20.9
Panagopoulou [25]	Greece	68 (49%)	19.81 ± 8.98	BMI < 10th percentile	45.5
Phong [21]	USA	49 (27%)	9.4 ± 5.2	BMI < 10th percentile	12
				MUAC*z* < −1.0	49
Poulimeneas [16]	Greece	84 (58%)	11.8 ± 3.9	BMI < 15th percentile	17.9
Wiedemann [27]	Germany	4557 (NR)	0–18	BMI < 15th percentile	30.4
Wiedemann [30]	Germany	2688 (NR)	0–18	BMI < 15th percentile	28.6
Zhang [29]	USA	13,021 (NR)	2–18	BMI < 15th percentile	10–30

BMI, body mass index; CF, cystic Fibrosis; MUAC, mid upper arm circumference; NR, not reported.
